# Scarlet Fever and the Board of Health

**Published:** 1877-06

**Authors:** Henry M. Lyman

**Affiliations:** Professor of Physiology and of Nervous Diseases in Rush Medical College—Chicago


### SCARLET FEVER AND THE BOARD OF HEALTH.

(*Read before the Chicago Medical Society, May* 7, 1877.)

By Henry M. Lyman, M. D.

[Professor of Physiology and of Nervous Diseases in Rush Medical College—Chicago.]

The current epidemic of scarlet fever does not seem to have in any respect added to the sum of our previous knowledge concerning the disease. The same tendency to increased mortality during the coldest months in the year; the same peculiar danger to children before their second dentition; the same malignity in communities which have long escaped the scourge —have all been again remarked. Some persons have tried to connect the unusual mortality with a corresponding uncleanly condition of the city; but I have often seen the city in as bad a condition without any noticeable fatality from scarlet fever. In fact, the portions of the city—on the West side, at least,—

which have this year been comparatively free from the disease, and which have been claimed as examples of the good results of drainage and paving, have during previous years been full of scarlet fever. As far as my experience goes, the disease has, in the better quarter of the West side, during the three years which were closed by the summer of 1876, been endemic until it well-nigh exhausted itself for lack of material. Nor has the weather during the past winter been of a nature calculated to increase the general mortality. Cold of an unusual uniformity, with a remarkable absence of south winds, so that the snow remained on the ground with scarcely any thaw from the first of December till the first of March, producing a degree of atmospheric dryness and purity seldom equaled in this region--such were the characteristics of the winter. Perhaps the only extraordinary condidition which might be recognized as widely operative, was the increased pressure of poverty, leading to greater crowding of the population into fewer and smaller rooms during the coldest months of the year; and yet, nearly all the worst cases which I have seen have occurred in families whose residence and habits of life have remained unchanged for several years.

In short, then, the sum and substance of our knowledge of scarlet fever is now just what it was twelve months ago, to wit: Scarlet fever is a disease which is within certain limits communicable from person to person. The contagious element can hardly be considered an aerial poison like malaria or like the poison of measles. It rather resembles the poison of typhoid fever in its limitation to a narrow sphere about the patient; yet there is no satisfactory evidence that it can be diffused by water in any way comparable to the diffusion of typhoid fever by liquids. It seems to occupy a rank between typhoid fever and syphilis, so far as its communicability is concerned. Consequently we find that it can under favorable circumstances be prevented from spreading through a family. I have never known it to pass from one house to an adjoining dwelling. I have never carried it home to my family, or to the families of any of my patients while attending even the most malignant cases. I have known a physician to get out of bed in the night, step across the hall into the room of a family under the same roof, where children were dying with the most virulent forms of the disease, and, after administering to their necessities, return immediately to his bed without communicating the disease to the child who was always his bed-fellow. In like manner, I have often seen the disease limited to the inmates of a single apartment in boarding-houses where several families lived under the same roof, and in tenement houses, and even in private residences where it was possible to isolate a child from the other members of the family. The contagious principle is not sufficiently aerial to diffuse itself through a large house; it must be actually carried from room to room in order to infect an entire domicile. In this respect my experience during the past year is identical with that of previous years.

As for the value of the ordinary disinfectants, I have no decided conviction. Their use is sometimes coincident with the restriction of the disease; but I have also seen it spread through a family in spite of their diligent use. Whether disinfectants may not be serviceable in prevention of contagion by transport of articles which have been in contact with the disease, is still an open question; consequently, I should incline to the safer side, and would join in recommendation of disinfectants. They certainly can do no harm.

As for the mortality of the epidemic, it has certainly been large. During the past twelve months I have lost six cases—eleven per cent. of the total number treated. Of these, five fatal cases occurred during the month of January—the month when the greatest mortality was to have been expected—and with but one exception they were children for whom any acute disease was to have been dreaded. Eleven per cent. is a good round figure; but when I include a series of years in my inquiry, I cannot consider scarlet fever a very mortal disease. Until the present year, a most insignificant per centage of fatal cases of scarlet-fever has been my usual experience—it was two per cent. last year—and I do not believe mine differs, in this respect, from that of many other physicians.

In this connection, it is much to be regretted that we cannot arrive at a satisfactory conclusion respecting the mortality rate of the present epidemic. The percentage of deaths from scarlet fever as compared with the mortality from other causes is easily obtained; but one need not look far between the lines of the recent report of the health commissioner to the Society of Physicians and Surgeons, to discover that he is not in possession of full returns from the epidemic. In view of the general interest of the community in the matter, and of the remarkable unanimity with which the members of the medical profession rallied to the support of the Board of Health, it is worth while to inquire into the cause of this undoubted fact.

When the physicians of Chicago assembled in council, on the 27th of Jan. 1877, their committee proceeded to report a document, copied at least in part, from the bulletins of the Boards of Health in Boston and New York, and containing a very judicious address to the public respecting scarlet fever and its general management. The report also contained a a statement of "What the Authorites ought to do," to the effect that they should,

1. "Require a report to the health department of every case of infectious disease.

2. "Officially inquire into the origin of disease in each case.

3. "Take such measures as will prevent communication between the infected location or house and those not infected."

Excellent so far. Nothing could be more thoroughly calculated to increase our knowledge of the *statistics* of scarlet fever, to say the least. But then the committee went on from generalities to particulars, of which the very first was most ingeniously calculated to neutralize in great measure, the previous recommendations;—they advised that upon every infected house a "placard" should be placed as a "warning to the public."

This novel device cannot have claimed for it any experience of its utility as a means of prevention of scarlet fever. It is a recent suggestion which originated as a mere proposal in the town of Christiania, Norway, and has been caught up without much consideration by some of the closet philosophers of the latest period. At first sight, the recommendation seems very plausible. Mark the house which contains scarlet fever; everybody will avoid the spot; the disease will therefore be limited to that dwelling, and the epidemic will soon die out. But in all natural sciences *a priori* speculations are of very little value. Experiment is the only test of the value of an hypothesis; consequently, I have watched with no little interest the course of this experiment. I have taken pains to make inquiries, where I have had no opportunity to make the experiment myself, and my convictions are based upon a pretty wide range of facts; though inasmuch as many of them have come to my knowledge in the shape of confidential communications, I cannot support my position by a citation of authorities and details, such as would be desirable in a strictly scientific document. In conversation with other physicians, I have often remarked a species of uneasiness with regard to the subject of scarlet fever, which is not without significance, and I presume that I have sometimes laid myself open to the suspicion of being a spy from the health office. If such be the case, I hope this paper may relieve me from any such reproach.

What, then, seems to be the practical result of the experiment? A very wide-spread and deep feeling of opposition to the display of placards upon infected houses. This feeling, ot course, has not yet invaded the whole community, because only a small portion of its families have thus far experienced the infliction of the nuisance in question. But in almost every case of its experience, the feeling is, to put it in the mildest way, a feeling of profound dissatisfaction. In the first place, the mere sight of a placard is often sufficient to throw a whole neighborhood of nervous ladies into paroxysms of most painful excitement. Strong men, even,—hard-hearted fellows of whom such a thing would never have been predicted—have told me that they could not endure the shock of meeting such a spectre at their door on returning home from business in the evening. There is no doubt that the maintenance of such a notice upon a house is often a very considerable addition to the sufferings of an especially afflicted family. Such feelings should not be disregarded without sufficient cause. In dealing with the community, as physicians, we have to deal with three great classes of people: Those who are well off in this world's goods, those who live in their shops where their business is conducted, and those of the laboring class who live in the little cottages which are so numerous in this city. Let us see how these different people regard the matter.

The better class of citizens who dwell in large houses with several apartments, are generally amenable to reason of a certain kind. Some of them are exceedingly timid, over anxious and hysterical about sickness. They readily comprehend the current theories about the communicability of scarlet fever, and will do anything rational to prevent the spread of the disease. They obey the counsels of their physicians, and can easily be led to overcome their instinctive repugnance to that degree of publicity, etc., etc., which is inseparable from a public notice of sickness in the house. Were they alone to be taken into consideration, the practice might easily be defended. Mrs. A. drives out, on a fine afternoon, to call upon her dear friend Mrs. B., who lives in a distant part of the city; sees a scarlet fever notice on that lady's door, and, wheeling short about, lashes her horses in the direction of home, with a heart full of gratitude to the board of health, which had saved her from running right into a whole hornet's nest of scarlet rash. A very pretty story—much affected by the partisan of the prevailing fashion; but here is the other side, drawn from my own observation. Scarlet fever occurs in the person of a small child—one of a large family in an elegant residence. Upon the mother—a feeble lady in poor health—devolves the principal care of the patient; but the house is large, the child is isolated from the rest of the family, the servants are well trained, and everything moves on quietly until the fourth or fifth day of the disease, when a sanitary inspector makes his appearance and proceeds to erect his card against the house. This is the signal for a general insurrection among the divinities of the kitchen. "What is the trouble, didn't you know we had scarlet fever in the house?" "Yes." "Are you afraid of it?" "Not a bit." "Why, then, do you insist upon leaving us now?" "Won't stay in a house with *that thing* on the door." And away they go—the outraged goddesses, *Vera incessu pat-*

*uit dea.* "Well," says the crest-fallen doctor, when he hears this sorrowful tale, "can't we find somebody else to help you? Girls are plenty now." "Yes," replies the disheartened mother, "there are plenty of girls, but they won't, one of them, come near the house while that card is on the door."

Now if there is any one thing in social life which is a little meaner than anything else, it is the doing of that which will take from the invalid mother of a large family, the help of her servants at a time when she most needs their assistance. You can all imagine the feelings of a kind hearted physician when he finds that he has played this scurvy trick upon one of his best patrons and friends. Very naturally, a paper placard is not a very durable affair under such circumstances, and no one but an ass would be too inquisitive regarding the character of the elementary forces which disintegrate such inscriptions.

Let us pass to the other extreme of the social scale.

Sickness has raged for several weeks among the "nine children at the breast," in a single-roomed shanty on Ashbox street. At length, it is decided by a council of toothless crones, that Dr. Robinson shall be invoked. He pronounces it scarlet fever, and a sanitary official proceeds to conceal the greater part of the front of the edifice with a "warning card." He then creates in the house a vile odor, with certain compounds, (an act which, however, should be pardoned when we consider who he is and whence he comes), informs the surrounding population that Dr. Robinson is a fool, who knows nothing about the proper treatment of scarlet fever, and disappears—forever, let us hope. Immediately there is an outbreak in the neighborhood, and the health commissioner is mentioned in terms of anything but flattering respect. The victims of his administration curse without stint, while the spectators simply laugh him to scorn, and the "d—d doctor" who reported the case, comes in for a full share of abuse. The hard horse-sense of the common people, penetrates right through the transparent pretense of limiting the spread of scarlatina by any such flimsy sham as a paper card. It is the old story of Mrs. Partington, keeping out the Atlantic ocean with a broom, as long as a ceaseless tide of children and other living creatures continues to ebb and flow in and around the infected dwelling. "If the disease is communicable, how can you shut it up with a card? If it is so contagious as you say, why don't it spread through all the houses around?"

The consequence is that the health officers and medical men generally, sink in the estimation of the great majority of the public, which becomes correspondingly callous and indifferent to the well-considered and certain utterances of the same authorities in other matters, about which there can be no difference of opinion. This point reached, the officials cease to control the course of events; they become simply the dust of the whirlwind. When the health commissioner is mentioned in connection with scarlet fever, the common people laugh, and say, "Oh, yes, he had to do something to earn his money, and probably he couldn't think of anything else but to stick up his card around the city." Such trifling with sacred matters is to be regretted, and its occasion should not be lightly afforded.

One more class remains to be considered—the small shopkeepers and other people who live in the houses where they toil for their daily bread. This is the class to which the great majority of physicians belong, and it therefore forms the portion of the community for which our sympathies should always be most easily aroused.

By this class of people the matter of placards is regarded not merely with disgust or derision, but with absolute terror. For such a person a scarlet fever notice means loss of business, loss of income—very likely, ruin itself. Just consider what might happen to any physician for example. Scarlet fever in the family, a placard on the door for six weeks or two months —longer even, perhaps. The doctor has not been more fortunate than his fellows in the profession. In the best of times he can hardly make the two ends meet, there are so many little mouths in the nursery upstairs. And now comes sickness, and that terrible notice on the door, warning people to beware of that house, to avoid its inhabitants, to keep them far from the rest of mankind for no one knows how long. That means a forced contribution of two hundred, five hundred, or a thousand dollars, taken from the pocket of a brother mortal who had hardly the where-withal to keep soul and body together in the best of times. Is this right? Surely not without some most serious cause for such conduct. Is it necessary? Let us see.

I once had scarlet fever in my own family, and that, too, when I lived in a little bandbox of a house where the isolation of the sick could only be of the most imperfect character. I saw the opportunity for an experiment, and I performed it without hesitation. Taking no other precautions but those suggested by the ordinary habits of personal cleanliness, I went about my work as usual. My practice was excellent; there were frequent calls from patients at my house, and I was constantly employed. I took pains to watch the families and the people with whom I was thus brought in contact, and in not a single instance did scarlet fever occur among them, nor did any of the ladies whom I attended in confinement manifest the slightest puerperal accident. This experience is by no means uncommon, and is sufficient to prove the gratuitous character of the assumption that scarlet fever necessarily converts a house into a centre of infection which must be shunned like a plague-spot. The successful results of confining the disease to a single room by isolation of the patient, show that an entire building need not be considered dangerous because there is scarlet fever in some portion of the edifice. Consequently, when the disease appears in a family under such circumstances, the board of health, instead of doing its best to ruin them financially, should bend its efforts to the work of showing the unfortunate parties how to make themselves as harmless as possible by the use of disinfection, and by the seclusion of the sick away from the portion of the house which is used for the transaction of business. It is not right to insist upon measures which will deprive a man of his livelihood, on the ground that it is for the good of the public, unless that public is willing to take in addition the only step consistent with justice, and to assume the support of that man as long as he submits to the infliction. "But," say the advocates of the crimson-card, "would you not place a card upon houses in which there is small-pox, and is it not as necessary in scarlet fever as in small-pox?" Certainly, I reply, I would placard a small-pox case, because there is no degree of parallelism between the two diseases. Small-pox is a disease which can easily be prevented. In a civilized community it only occurs as the result of ignorance or carelessness,—two vices which merit all the punishment which can grow out of the disease and the isolation which it entails. Small-pox, moreover, is caused by a rather volatile poison which impregnates an entire dwelling, and a considerable atmospheric zone around it. But such is not the case with scarlet fever. Only a limited sphere is occupied by its infection, so that a house is by no means necessarily to be avoided because it may in some apartment contain scarlet fever. The disease is, also, one that cannot be prevented by any known measure, like vaccination, which is within the reach of every one. Families afflicted with scarlet fever, are therefore to be regarded with sympathy and compassion instead of being made the objects of punitive measures. And since the disease is only seldom dangerous to life, it cannot be worth while to attempt on its account to revive in the community the horrors of isolation and desertion which before the days of vaccination made an epidemic of small-pox such a terror to mankind. It is the dread of such isolation which causes much of the repugnance which people feel in connection with these obnoxious placards. Already, the first question is, "Must we have a card on the house?" Already I have been forewarned that if scarlet fever should invade such and such families, their choice of a physician would be determined by his conduct in the matter of reporting scarlet fever to the health office. Already there is in many quarters a reluctance to call in medical aid, for fear of the inevitable red card. More than once I have been consulted at my office about patients for whom a visit was not desired, but which I had reason to suspect were cases of scarlet fever. And this is only the beginning of the storm of dissatisfaction which is gathering, yet so enamored are the officials with their little two-penny game of cards, that I am told they will even fly at a case of measles, and cover it all over with pasteboard. This is certainly the *reductio ad absurdum* of the whole thing.

Nevertheless, all these inflictions might be justified, and might be tolerated by a long-suffering public, if it could be shown that they accomplished a single good result. On the contrary, there is not the slightest evidence of any value to show that the progress of the epidemic has been in the smallest degree affected by these odious notices. They have only served to irritate the public and to annoy physicians. This is the principal cause which has operated to prevent the health officers from getting full returns from the epidemic, so that one of the finest opportunities for the study of the disease has been only partially improved. The officials have thus by their conduct laid themselves open to the charge of being urgent about trifles, and of being negligent in matters of real importance. The rank and file of the profession are dependent upon the good will of their patrons, and they cannot afford to take sides in behalf of a measure which is both unpopular and useless. Consequently, I find everywhere great caution in the matter of diagnosis. The prudent physician makes a second visit before he communicates with the board of health. In doubtful cases, the patient gets the benefit of the doubt. Harmless rashes are of more frequent occurrence. Even the most enthusiastic admirers of the placard confess to having cases of roseola and the like which they do not report at the health office. We are all familiar with the fact that certain physicians always magnify their cases. Under their care tonsillitis becomes diphtheria, while intestinal catarrh assumes the dangerous form of Asiatic cholera. But now the tendency is all the other way; the endeavor is to minimize everything which looks like scarlet fever, so that it really begins to seem as if the current epidemic would finally resolve itself into a fiery cloud of red-gum and prickly heat. In point of fact, so far as any good result is concerned, the whole matter of placarding scarlet fever is, to use the words of one of our oldest, wisest, and best known physicians, *mere boyish play.*

In all these criticisms, however, I do not wish to reflect injuriously upon the worthy gentlemen who discharge the duties of the health office. They have honestly attempted to carry out the recommendations of the committee of physicians. They have had the courage to undertake the performance of an exceedingly unpopular experiment, and we should blame not them, but the feather-heads who originated the device. Our commissioner of health has displayed a laudable courage, and it is not too much to hope that he will also display an equally laudable discretion. A little patience, a little ingenuity, a little study of human nature, will accomplish far more good results than can ever be secured by harassing the profession and the public with law-suits and fines. We are too apt to admire the resort to force as a remedy for evils which we have not wit enough to control. Just as it is the most natural thing in the world for a cheerful doctor of divinity, surrounded by a fashionable congregation, to insist that the law should compel every citizen to pass his Sundays in a clean shirt, with a pitcher of cold water at his elbow, and a copy of the *Northwestern Christian Advocate* in his hand, —so it is exceedingly easy for a comfortable official, meditating in the seclusion of a governmental bureau, to think that everybody ought to overcome his natural feelings, and to submit without a murmur to any new-fangled experiment which certain hard-hearted doctors may see fit to try upon him. People who have been accustomed to ecclesiastical or military methods of administration will readily fall in with this way of thinking. But by the mass of mankind such methods have always been justly deemed vexatious, ineffectual, intolerable. If anything be true of our American institutions, it is their foundation in an abiding protest against priestcraft and military domination. No methods of procedure which ignore this fact can for a moment stand in the face of public opinion. Hence it behooves our servants if they are wise, to find out where they stand, and to BEWARE, lest they bury themselves deeper than the ninth hell of the Inferno.

